# Endothelial Piezo1 channels as sensors of exercise

**DOI:** 10.1113/JP274396

**Published:** 2018-01-09

**Authors:** David J. Beech

**Affiliations:** ^1^ Leeds Institute of Cardiovascular and Metabolic Medicine, School of Medicine University of Leeds Leeds UK

**Keywords:** non‐selective cationic channel, endothelium, shear stress, resistance artery, exercise

## Abstract

Piezo1 channels are newly discovered ion channels which have come to the fore as players in endothelial biology. They have a key role as sensors of shear stress, a frictional force which arises in vascular biology because of blood flow. Endothelial Piezo1 channels are critical in murine embryonic development, just after the heart starts to beat and drive blood into the nascent endothelial network. In contrast they are not critical at the adult stage but they are important for performance in whole body physical activity where they have a vascular bed‐specific effect to cause mesenteric resistance artery vasoconstriction, achieved through opposition to the vasodilatory mechanism of endothelium‐derived hyperpolarization. These are our first insights into the relevance of endothelial Piezo1 channels and there is clearly much more to be done to understand the significance and underlying molecular mechanisms. The suggestion that they constitute an exercise sensor by virtue of their shear stress‐sensing capability is intriguing and could open the way to better understanding of the molecular basis of the response to exercise and determination of how the health benefits of exercise arise. Enhanced Piezo1 channel activity has been demonstrated in response to the Yoda1 molecule and this suggests the possibility for developing tools which probe and manipulate this aspect of the exercise system. Whether such agents might progress to ‘exercise pills’ and whether the existence of such pills would be desirable are matters for further work and debate.

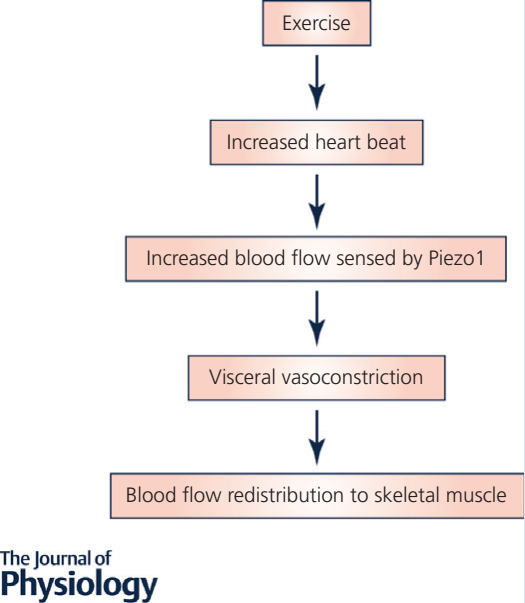

## Introduction

Endothelial cells form the inner lining of blood vessels and the interface between the blood and the vascular wall and tissue beyond. They are critical in physiology and disease. Routinely these cells experience and respond to a range of mechanical forces which include those arising because of blood flow, vascular remodelling, injury, and physical movement of the body or its parts. A particularly important force is the frictional force of shear stress, arising because of blood flow (Chiu & Chien, 2011). Shear stress is actively sensed by endothelial cells in ways which enable vascular development and which usually maintain an efficient and healthy vasculature. The mechanisms by which endothelial cells sense shear stress are gradually becoming known to us (Chiu & Chien, 2011; Conway & Schwartz, 2012; Ando & Yamamoto, 2013; Baratchi *et al*. 2017; Hyman *et al*. 2017). We have suggested that Piezo1 channels are key components of the system, acting as force sensors and transducers of force into biological change (Li *et al*. 2014; Hyman *et al*. 2017; Rode *et al*. 2017).

## Importance of Piezo1 channels as sensors of shear stress

Piezo1 channels are ∼25‐pS Ca^2+^‐ and Na^+^‐permeable channels of the plasma membrane (and possibly other membranes). They were discovered in a targeted short interfering RNA screen against mechanically‐activated ionic currents in the N2A neuroblastoma cell line (Coste *et al*. 2012). This discovery might not seem relevant to the sensing of shear stress but it turns out to be important. It is well established that Ca^2+^ entry is one of the earliest events caused by shear stress and a shear stress‐sensitive ∼25 pS non‐selective cationic (Ca^2+^‐ and Na^+^‐permeable) channel was described some time ago in endothelial cells but the molecular basis was not determined at the time (Brakemeier *et al*. 2002). The unitary conductance of Piezo1 channels was tantalising because other candidate shear stress‐sensing Ca^2+^‐permeable channels have quite different conductances (Hyman *et al*. 2017). We therefore investigated Piezo1 in the endothelial context. We found that Piezo1 channels had profound importance for shear stress‐evoked Ca^2+^ signalling and non‐selective cationic channels in mouse and human endothelial cells (Li *et al*. 2014). They were able to reconstitute shear stress sensitivity in human embryonic kidney (HEK) 293 cells which were otherwise resistant to shear stress (Li *et al*. 2014) and we went on to show that endogenous channels of the endothelium were activated by shear stress in excised outside‐out cell‐free membrane patches, suggesting that the channels are likely to be direct sensors of shear stress or a closely linked force (Rode *et al*. 2017).

## Embryonic lethality of *Piezo1* gene disruption

**Figure 1:**
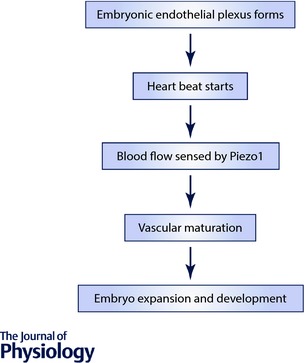
Piezo1 channels and vascular maturation Summary of the hypothesis that Piezo1 channels are blood flow sensors which are critical for vascular maturation in murine embryogenesis (Li *et al*. 2014).

Global and endothelial Tie2‐driven Piezo1 knockout in the mouse was embryonic lethal just at the time when the vasculature matured in response to shear stress, from embryonic day 9.5 onwards (Li *et al*. 2014). Endothelial cells were present in knockouts but vascular maturation to larger vessels was disrupted (Fig. 1). Pathway analysis pointed to downstream calpain regulation of focal adhesion turnover as a mechanism to release endothelial cells for reorganisation (Li *et al*. 2014). Subsequent human genetic association studies suggested importance of Piezo1 in human vascular development (Fotiou *et al*. 2015).

## Conditional *Piezo1* gene disruption in endothelium of the adult mouse

Because of the embryonic lethality in constitutive Piezo1 knockouts, investigation of Piezo1 in the adult required conditional disruption delayed until after mice had matured. Disruption at this stage, specifically in the endothelium, was not lethal; these mice turned out to be relatively normal (Rode *et al*. 2017). However, when we studied freshly isolated endothelium from second‐order mesenteric resistance arteries we observed striking constitutive activity of endogenous channels which could be further enhanced by shear stress and which were totally dependent on Piezo1. This channel activity was robust, maintained even in the sparse environment of the outside‐out patch (Rode *et al*. 2017). This suggested to us that the conditional Piezo1 knockout mice should have a phenotype.

## Anti‐EDH effect of endothelial Piezo1 channels

In an effort to identify the phenotype we first performed contraction studies of second‐order mesenteric resistance arteries in the absence of shear stress. Superficially these arteries were normal, showing contractile responses to phenylephrine and endothelium‐dependent relaxation to acetylcholine. However, although there was no significant difference compared with controls, we got the impression that the response to acetylcholine might be a little larger. We looked more closely at this acetylcholine response because it is known to be mediated by multiple mechanisms which might compensate for each other. This closer inspection led us to find that Piezo1 channels were opposing the component of the response attributed to endothelium‐dependent hyperpolarization (EDH) (Garland & Dora, 2017), consistent with constitutively active Piezo1 channels acting as an ‘anti‐EDH’ (Rode *et al*. 2017).

Because EDH is a relaxation mechanism, a contractile function of Piezo1 channels was suggested, which might seem surprising in view of prior positive associations of endothelial Piezo1 channels with nitric oxide synthase activity, which has relaxant capability (Li *et al*. 2014; Wang *et al*. 2016). Nevertheless it is important to bear in mind that Piezo1 channels are both Na^+^ and Ca^2+^ permeable. Entry of cations is depolarizing, so it should oppose EDH and promote contraction. Entry of Ca^2+^, a key signalling ion, can activate many mechanisms, one of which is nitric oxide synthase, so there is potential to stimulate nitric oxide production and thus promote relaxation. Piezo1 channels therefore present a dichotomy for endothelial biology, having the potential to cause opposing effects – contraction and relaxation (Rode *et al*. 2017). Whether molecular mechanisms exist to favour one mechanism over the other, perhaps in a vascular bed‐specific manner (see below), remains to be determined. Nevertheless we asked the question: how does this dichotomy play out in the whole animal?

## Endothelial *Piezo1* disruption specifically blunts blood pressure elevation in whole body physical exercise

**Figure 2:**
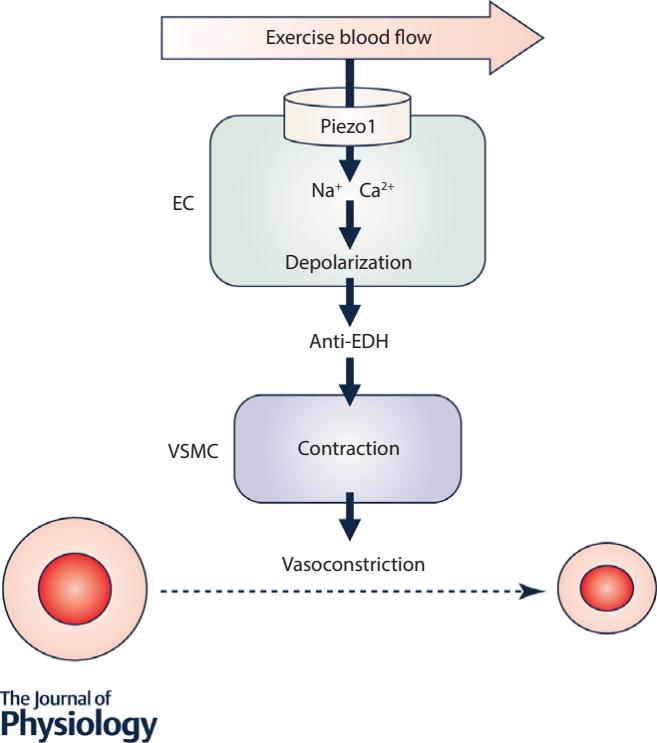
Activation of Piezo1 channels Summary of the hypothesis that Piezo1 channels are blood flow sensors which are activated by the increased blood flow of whole body physical activity and thereby cause vascular bed‐specific (e.g. mesenteric) vasoconstriction by opposing endothelium‐dependent hyperpolarization (EDH) in adult mice (Rode *et al*. 2017). EC, endothelial cell; VSMC, vascular smooth muscle cell.

Measurement of blood pressure in freely moving conscious mice revealed a striking role of endothelial Piezo1 (Rode *et al*. 2017). While it lacked significant effect when mice were physically inactive, it became important when mice were running voluntarily on a wheel – i.e. during whole body physical exercise. The elevated systolic and diastolic blood pressures seen during exercise were strongly blunted by Piezo1 deletion, suggesting that a Piezo1‐dependent vasoconstrictor mechanism was activated by the exercise (Rode *et al*. 2017). It has long been suspected that endothelium contains exercise sensors and that what is being sensed is the increased blood flow of exercise (Joyner & Casey, 2015). What we suggest is that Piezo1 channels confer such a sensing mechanism in mesenteric resistance and other arteries which constrict during exercise (Rode *et al*. 2017). We provided evidence that the mechanism of this effect is shear stress‐induced activation of Piezo1 channels which depolarises the endothelial cells, which then depolarises the adjacent vascular smooth muscle cells that are electrically coupled via gap junctions, which in turn activates L‐type voltage‐gated Ca^2+^ channels which are expressed in vascular smooth muscle cells and are well established to trigger vascular smooth muscle contraction and thus vasoconstriction (Fig. 2). We could clearly demonstrate such a mechanism in mesenteric resistance arteries *ex vivo* (Rode *et al*. 2017).

## Anti‐EDH effect of endothelial Piezo1 channels is vascular bed specific

Whole body physical activity is not only associated with vasoconstriction. Vasoconstriction in exercise is restricted to organs that are relatively unimportant during the periods of physical activity, such as the intestines. In skeletal muscle there is vasodilatation. That is, there are vascular bed‐specific responses to exercise that enable redistribution of blood flow (Joyner & Casey, 2015). The changes in blood pressure reflect total peripheral resistance and other determinants such as cardiac output. Importantly, when we performed contraction studies on saphenous artery, we found no anti‐EDH effect of Piezo1 (Rode *et al*. 2017). Saphenous artery supplies the leg and is similar in calibre to second‐order mesenteric artery. Carotid artery also lacked the anti‐EDH effect (Rode *et al*. 2017). Therefore there is vascular bed specificity of the Piezo1 vasoconstrictor mechanism, suggesting that it is important for redistributing blood flow in exercise, enhancing performance by restricting blood flow to the intestines and enabling its redirection to skeletal muscle.

Prior study of rat arteries suggested dominance of the EDH mechanism in mesenteric artery but absence in femoral artery (Sandow *et al*. 2002). An identified mechanistic difference was the presence of efficient gap junction communication between endothelial cells and smooth muscle cells in mesenteric but not femoral artery (Sandow *et al*. 2002). This could be a simple mechanism by which flow‐activated Piezo1 channels could achieve vasoconstriction in one artery but dilatation in another; Piezo1‐mediated depolarisation causing smooth muscle depolarisation (and thus contraction) in the gap junction‐coupled artery but only impacting smooth muscle through enhanced nitric oxide production in the non‐coupled artery. We observed a small EDH component of vasodilatation in mouse saphenous artery, however (Rode *et al*. 2017). Therefore additional mechanisms may impact such as vascular bed‐specific distribution of functional Piezo1 channels.

## Disruption of endothelial *Piezo1* causes reduced exercise performance

The idea that Piezo1 channels confer a vascular bed‐specific vasoconstrictor effect that is advantageous for whole body physical performance was supported by careful analysis of physical activity of mice on a running wheel (Rode *et al*. 2017). We found that the number of bouts of physical activity, the distance run and time active were all significantly reduced in the absence of endothelial Piezo1 (Rode *et al*. 2017). Moreover the knockout mice lost more weight during exercise – equivalent in magnitude to an 80 kg human losing about 2 kg in a week (Rode *et al*. 2017). The mechanism of the latter effect is not yet clear but it might be explained by the mice expending more energy in an effort to deliver performance. It was also intriguing that the compromised performance of the knockouts was lost after 1 or 2 nights of exercise, suggesting that compensatory mechanisms were induced (Rode *et al*. 2017).

## A small molecule (Yoda1) mimics the effect of shear stress on Piezo1 channels

Screening of a library of 3.25 million chemicals against cells overexpressing Piezo1 led to the discovery of a low molecular weight synthetic chemical which activates Piezo1 channels (Syeda *et al*. 2015). This compound was named Yoda1, which nicely relates to the Yoda character of the Star Wars films. The Yoda1 discovery was not made by us but we went on to synthesise Yoda1 and studied its effects on overexpressed Piezo1 and endogenous Piezo1 of the endothelium (Rode *et al*. 2017). Yoda1 lacks potency and has relatively poor solubility but it is nevertheless a useful and apparently specific activator of Piezo1 channels. Importantly it nicely mimicked effects of shear stress on endothelial cells (Rode *et al*. 2017). Therefore there would seem to be an agonist binding site on or near to Piezo1 channels which enables enhanced channel activity.

## The concept of an exercise pill

Although Yoda1 is a tool compound which is unsuitable for *in vivo* studies, our findings suggest that it might be possible to chemically induce or enhance Piezo1 channel activity and thereby potentially enhance benefits of exercise (Rode *et al*. 2017). At the moment, this is a hypothesis and vision for future work. We need improved Yoda1‐like compounds to test the idea, and complexities of the biology will need to be understood if we are to properly design the experiments. One obvious challenge is the expression of Piezo1 in other cell types and tissues where its roles may be unrelated to exercise; another is the possibility for Piezo1 channels to stimulate nitric oxide production; another is the apparent vascular bed specificity of Piezo1 channel function, suggesting that a chemical activator of Piezo1 channels would need to also act in a vascular bed‐specific manner if it is to mimic effects of exercise. Nevertheless it is highly likely that effects of exercise (whether impacting performance or health) are mediated through molecular mechanisms which are amenable to chemical modulation. Therefore, it should be possible, through appropriate knowledge and research, to develop an ‘exercise pill’. Should such a pill be developed, it will be important to consider carefully how it should be used and how it might impact our lives.

## Debate on the rights and wrongs of working towards an exercise pill

Physical exercise is known to have powerful health benefits against cardiovascular disease, diabetes and other major diseases of our modern societies (Lavie *et al*. 2015). It very often delivers this benefit without obvious adverse effects, aside from occasional physical injuries. The possibility to benefit in this way – through regular physical activity – already exists for many people and it is their choice and the choice of societies whether and how to take advantage of it. However, it is also true that many people cannot exercise or cannot exercise sufficiently; for different reasons which include physical injury, disability, illness, demanding sedentary employment, old age and natural physical or mental dispositions against physical exercise. These people often are not able to achieve the health benefits of exercise. For these people, an ‘exercise pill’ could be beneficial for their health and be ethically justifiable, particularly if used to enhance the benefits of moderate exercise and if used in conjunction with protocols which support the retention of muscle mass and positive psyche. Should we try to achieve such medication? There is a view that working to generate an exercise pill is like doing research to encourage people to smoke because it might encourage people not to exercise, but this is a narrow perspective when there are people who really cannot exercise and when none of our societies currently achieves a culture in which all exercise‐capable individuals exercise sufficiently throughout their lives. We should encourage debate of this matter not only because of our Piezo1 findings but also because of knowledge of other aspects of the exercise system such skeletal muscle glucose uptake (Richter & Hargreaves, 2013) which suggest, unsurprisingly, that the exercise system is a molecular system which is within our capabilities to understand. We will inevitably work towards this understanding, so a question will be: what shall we do with this information?

## Conclusions

We conclude that Piezo1 channels are important in endothelial biology, embryonic development and vascular homeostasis in whole body physical exercise and that the central molecular purpose of the channels in the endothelium is to be transducers of the frictional force of shear stress, a force arising because of blood flow. There remains much to understand about this biology and its relevance to disease, but the functional importance of Piezo1 suggests that the effort will be worthwhile.

## Additional information

### Competing interests

None.
